# Masticatory function parameters in patients with removable dental prosthesis

**DOI:** 10.25122/jml-2019-0028

**Published:** 2019

**Authors:** Alexandra Melania Oncescu Moraru, Cristina Teodora Preoteasa, Elena Preoteasa

**Affiliations:** 1. Department of Complete Denture, Faculty of Dental Medicine, “Carol Davila” University of Medicine and Pharmacy, Bucharest, Romania; 2. Department of Scientific Research Methods-Ergonomics, Faculty of Dental Medicine, “Carol Davila” University of Medicine and Pharmacy, Bucharest, Romania

**Keywords:** mastication, chewing, food, carrot test, edentulism, denture

## Abstract

This study aimed to assess the masticatory efficiency in patients with a removable dental prosthesis, presenting different systemic, oral and prosthetic states while chewing different foods. The study was conducted on a convenient sample of patients aged 45 and above, with removable prostheses in at least one jaw. Patients were asked to chew samples of digestive biscuits, apple, and carrot, until the sensation of swallowing. The recorded masticatory function parameters were: chewing time, the number of mastication cycles, mean masticatory cycle duration, and chewing frequency. We found out that the masticatory functional parameters registered statistically significant differences according to the chewed food (e.g., generally the highest values were recorded for carrot and lowest for apple), most likely this being in relation to food’s consistency, wetting, and adherence. High positive correlations were found between the chewing time and the number of mastication cycles for all three foods taken into consideration. Higher values for chewing time and number of mastication cycles were found for all foods in patients with complete dentures and overdentures, and while chewing carrot in patients with altered general status and of advanced age. Therefore, it that it takes a different time and number of mastication cycles to complete chewing, in relation to individual and food characteristics, to the systemic, oral and prosthetic patient’s status. The residual teeth number and the type of prosthetic rehabilitation favor the adaptation and improvement of masticatory parameters and can have marker value for masticatory efficiency.

## Introduction

Mastication is the first stage of initiation in the digestive function, an essential function through which nutrient intake necessary to the body for the health and well-being of humans is provided. Thus, mastication is a primary oral function, which is directly related to the general health status and the quality of life of the individual.

Present since birth, mastication is considered to be the first oral function. It evolves in the course of life through the development and evolution of the dental-maxillary apparatus, but also in relation to the diversification of nourishment, with different types of food. Therefore, if newborn food is taken in through sucking (the foods being liquid, such as milk or tea), gradually, through oral changes and primordially through teeth eruption, the possibilities of food processing increase and so does its consistency. Thus, mastication becomes a complex biomechanical process characterized by the crushing and trituration of food, helped by teeth, through the action of the mobilizing muscles of the mandible as well as other head muscles (lips, cheeks, tongue). Foods are broken down into small fragments, which are saturated with saliva to form the food bowl, followed by swallowing and passing it to the next segment of the digestive tract, the esophagus.

In the course of human development and mastication, as an automatic function, within the framework of this function appear the reflex circuits that explain the complex masticatory process, with the harmonious participation of different anatomical structures (teeth, periodontium, muscles, jaws, temporomandibular joint, vascularization, nerve endings and so forth). As a biomechanical process of oral digestion, teeth participate in mastication, and after their loss, their substitutes (the different types of dental prosthetic restorations) have different functional characteristics from those of the anatomical structures.

Mastication takes place through a repetitive process of moving the mandible relative to the jaw during masticatory cycles, which are related to individual characteristics, oral status and food characteristics (type and consistency of the food). During a masticatory cycle, there are several movements, namely: first, the mandible moves away from the jaw so that the food fragment is introduced in the mouth, then during the second movement the mandible approaches the jaw and follows the third movement when the antagonistic teeth establish contact through nourishment. A new masticatory cycle begins by opening the mouth, then closing it and crushing the food. Several such masticatory cycles take place until the end of the process of crushing food, forming the food bowl through the use of saliva, swallowing it, passing it into the oropharynx and further into the esophagus.

There are various factors that may affect masticatory efficiency, including: the condition of the teeth, the appearance of the antagonists and the number of mastication units, the size and the relief of the dental surfaces involved in the masticatory process, the masticatory movements, the chewing tempo, aspects related to prosthetic rehabilitation, consistency and size of the food fragments relative to their preparation adapted to the swallowing capacity of the individual [1,2].

The aim of the study was to assess the masticatory efficiency in patients with a removable dental prosthesis, presenting various systemic, oral and prosthetic states, while chewing different foods.

## Materials and Methods

The study was conducted on a convenience sample of patients over 45 years of age, having at least one removable dental prostheses in one of the jaws. Complete and partially edentulous patients, restored by complete dentures, overdentures on teeth, implant overdentures, and removable partial prostheses in at least one jaw were included. Considering that patients with different conditions were included in the study, as well as their age, various comorbidities should be expected. However, patients with unrestored edentulism and patients with altered systemic status, with considerable communication deficits or mastication deficiencies, were excluded from the study.

For each patient included in the study, three types of mastication tests were performed, namely for three types of food. The duration and the number of mastication cycles were determined during mastication of digestive biscuits, carrot, and apple, and in the end, the mastication frequency and the mean duration of a masticatory cycle were calculated.

To perform the tests, food samples of each type of food, weighing 10g for carrot and apple and 5g for the biscuit, were taken. The mastication was performed until the sensation of swallowing occurred. For each of these tests, the time of mastication (recorded in seconds) and the number of mastication cycles up to the swallowing of the food bowl were determined. In addition, the mean duration of a chewing cycle was calculated as the ratio between the time of chewing and the number of mastication cycles for each recorded sample. The chewing frequency was obtained by the ratio of the number of cycles to the duration of chewing until swallowing [3].

Also, general data concerning patients and their oral and prosthetic status (age, sex, edentulism type, type of dental prosthesis, general disorders) were recorded.

The statistical analysis took into account its purpose, the variables type, and for the quantitative variables the normality of their distribution. Non-parametric tests were used, as variables were not normally distributed.

The Friedman test was used to see if there is a statistically significant difference between dependent variables (masticatory function parameters) while chewing apple, carrot, and biscuit. Wilcoxon signed-rank tests were used as post-hoc tests to see if the difference occurs between a pair of two foods. Post-hoc analysis was conducted with a Bonferroni correction, namely as significance level initially used was 0.05, the Bonferroni correction was calculated by dividing it by 3 (corresponding to 3 repeated measurements, i.e., for the biscuit, carrot, apple), resulting in a new significance level of p=0.05/3 = 0.017 that was used.

The Spearman test was used as a correlation test. The Mann-Whitney U test was used to compare two unpaired variables.

SPSS Statistics software was used for statistical analysis. The threshold of statistical significance was set at p <0.05.

## Results

The study was conducted on 34 patients who performed all three chewing tests, more precisely for a digestive biscuit, apple, and carrot. Patients included in the study were aged 49–90, with a mean age of 68. Most of the subjects included in the study were women (n=20), and the rest of them were men (n=14).

**Masticatory function parameters according to food type.** There were found statistical differences between the mastication of various food samples regarding the parameters of the masticatory process recorded in this research. The highest values of mastication time, the number of mastication cycles and the mean duration of a masticatory cycle were for carrot, followed by the values for biscuit mastication, the lowest values being recorded for apple mastication (Table 1). There was found a statistically significant difference for chewing time up to the appearance of swallowing sensation among mastication of the biscuit, apple or carrot, Friedman test χ^2^(2)=68.00, p<0.001. There were statistically significant differences between chewing time for all pairs of food taken two by two, between apple and biscuit (Z=–5.120; p<0.001), between carrot and biscuit (Z = –5.105, p <0.001) between carrot and apple (Z = –5.120; p <0.001).

**Table 1:** Parameters of the masticatory cycle for biscuit, apple and carrot

Parameters of the masticatory cycle	Food	Mean	Median
Chewing time (seconds)	Biscuit	20.76	21
	Apple	14.06	14
	Carrot	27.88	27
Number of masticatory cycles	Biscuit	32.21	31
	Apple	24.29	24
	Carrot	37.79	37
Mean duration of masticatory cycle (seconds)	Biscuit	0.66	0.66
	Apple	0.57	0.58
	Carrot	0.73	0.72
Chewing frequency	Biscuit	1.50	1.50
	Apple	1.73	1.71
	Carrot	1.35	1.37

There was also a statistically significant difference between the number of masticatory cycles performed up to the perception of the swallowing sensation between the biscuit, apple and carrot, Friedman test χ^2^(2)=68.00, p<0.001. Statistically significant differences were observed between the number of chewing cycles for all pairs of food taken two by two, between apple and biscuit (Z = –5.120, p <0.001), between carrot and biscuit (Z = –5.120, p <0.001) , between carrot and apple (Z = –5.129; p <0.001).

There was a statistically significant difference in duration of a masticatory cycle recorded in seconds among the masticatory situation for the biscuit, apple or carrot, Friedman test χ^2^(2)=68.00, p<0.001. There were statistically significant differences between the number of mastication cycles for all pairs of food taken two by two, between apple and biscuit (Z = –5.087; p <0.001), between carrot and biscuit (Z = –5.087; p <0.001), between carrot and apple (Z = –5.087; p <0.001).

A statistically significant difference between chewing frequency in the biscuit, apple, or carrot mastication was observed, Friedman test χ^2^(2)=68.00, p<0.001. There were statistically significant differences between the number of mastication cycles for all pairs of food taken two by two, between apple and biscuit (Z = –5.088; p <0.001), between carrot and biscuit (Z = –5.087; p <0.001), between carrot and apple (Z = –5.087; p <0.001).

**Figure 1:**
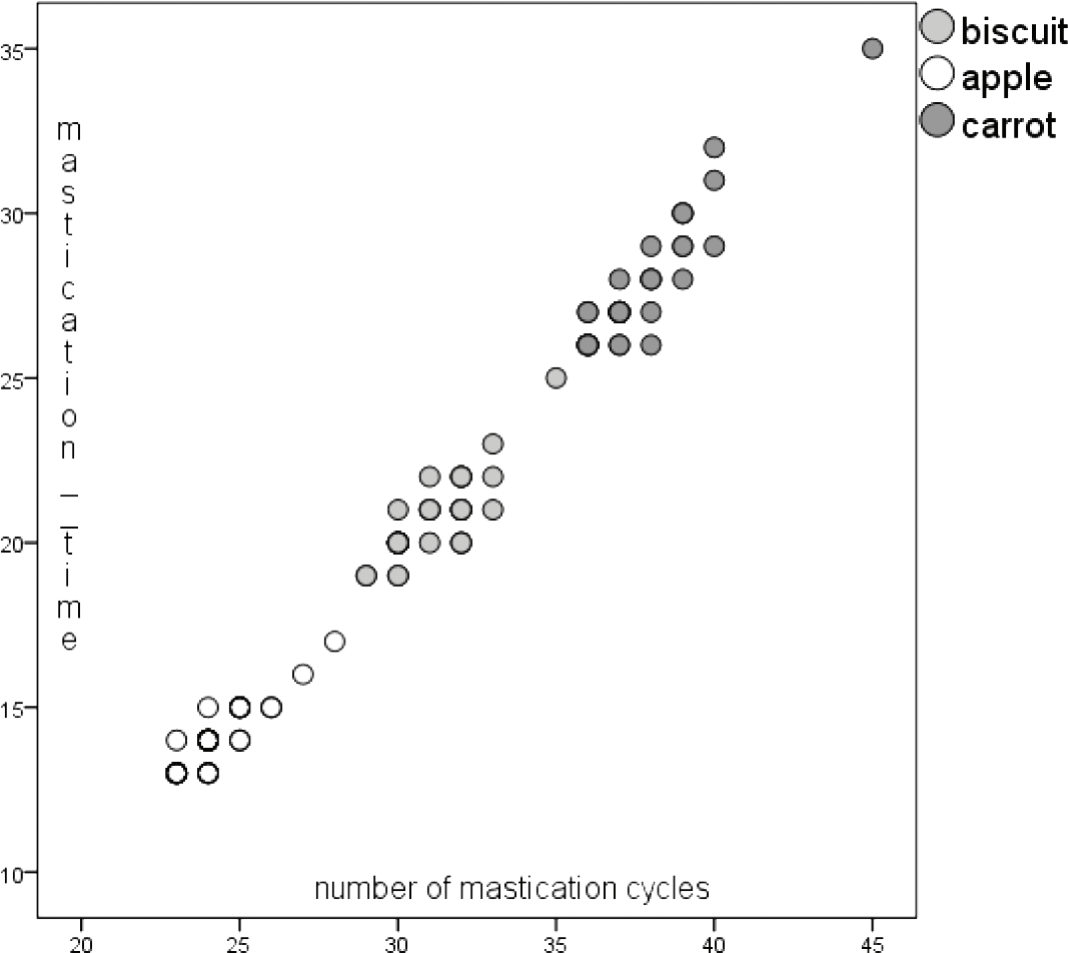
Figure 1: Correlation between mastication time and the number of mastication cycles

Using the Spearman test, there were high positive correlations found between the chewing time and the number of mastication cycles for all three foods taken into consideration: for the biscuit (correlation coefficient = 0.760, p <0.001); apple (correlation coefficient = 0.832; p <0.001); carrot (correlation coefficient = 0.886; p <0.001) (Figure 1).

**Masticatory function parameters according to the type of dental prosthesis.** Depending on the type of dental prosthesis, it was analyzed using the Mann-Whitney U test if there was statistically significant difference between complete edentulous patients or those with very few residual teeth (restored with complete dentures, with teeth or implant overdentures; n=16) and patients with more residual teeth (restored with partial prostheses, n=18). It has been observed that the time of mastication and the number of mastication cycles until swallowing were significantly higher in the patients with complete dentures or overdentures. The mean duration of a chewing cycle and chewing frequency were statistically significantly different only for the apple (Table 2).

**Table 2:** Parameters of mastication for clinical situations of edentate and prosthesis patients

Parameters of masticatory cycle	Food	Complete dentures or overdentures		Partial dental prosthesis		p
		Mean	Median	Mean	Median	
Chewing time (seconds)	Biscuit	21.25	21	20.33	20	0.021*
	Apple	14.63	15	13.56	13.5	0.001*
	Carrot	28.75	28.5	27.11	27	0.015*
Number of masticatory cycles	Biscuit	31.81	32	30.67	30	0.009*
	Apple	24.94	28.75	23.72	24	0.002*
	Carrot	38.5	38.5	37.17	37	0.021*
Mean duration of masticatory cycle (seconds)	Biscuit	0.66	0.66	0.66	0.66	0.564
	Apple	0.58	0.58	0.57	0.56	0.018*
	Carrot	0.74	0.74	0.72	0.72	0.079
Chewing frequency	Biscuit	1.49	1.5	1.50	1.50	0.564
	Apple	1.70	1.71	1.75	1.76	0.018*
	Carrot	1.34	1.34	1.37	1.37	0.079

**Masticatory function parameters according to systemic status.** As the systemic status, the patients presented themselves as follows: 11 were clinically healthy while the rest presented various general health conditions (12 with cardiovascular diseases, 2 with diabetes, 4 with digestive diseases, 3 with tumor diseases, 3 with neuropsychiatric diseases and 4 with other ailments). Of these patients, 10 were diagnosed with two general ailments and 4 of them with three general affections. Statistically significant differences between those with healthy clinical status and those with various diseases were observed only for the carrot, where the time of chewing, the number of mastication cycles and the mean duration of a chewing cycle were lower in those with a general healthy clinical status, while chewing frequency was higher for them *(Table 3).*

**Table 3:** Parameters of mastication according to the general status

Parameters of masticatory cycle	Food	General status – clinically healthy		General status – altered		p
		Mean	Median	Mean	Median	
Chewing time (seconds)	Biscuit	21.18	20	21.04	21	0.061
	Apple	13.82	14	14.17	14	0.437
	Carrot	26.45	26	28.57	28	0.001*
Number of masticatory cycles	Biscuit	30.82	30	31.39	31	0.274
	Apple	24.09	24	24.39	24	0.613
	Carrot	36.55	36	38.39	38	0.001*
Mean duration of masticatory cycle (seconds)	Biscuit	0.65	0.65	0.67	0.66	0.071
	Apple	0.57	0.58	0.58	0.58	0.409
	Carrot	0.72	0.72	0.74	0.73	0.021*
Chewing frequency	Biscuit	1.52	1.52	1.49	1.50	0.071
	Apple	1.74	1.71	1.72	1.71	0.409
	Carrot	1.38	1.38	1.34	1.35	0.021*

**Masticatory function parameters according to sex and age.** There were no statistically significant differences between women and men on the analyzed parameters of mastication. Positive correlations were observed between age and biscuit mastication time (weak linear relationship, r = 0.346, p = 0.045), carrot mastication time (average linear relationship, r = 0.576, p <0.001) and number of mastication cycles in carrot (average linear relationship, r = 0.621, p <0.001). In other words, with increasing age, there was a tendency to increase the value of those parameters of mastication.

## Discussions

The results of this study suggest that there are differences between the parameters of the chewing cycle depending on the type of food, most likely in relation to its consistency, the degree of softening (wettening) and the adherence of the foods. There is also a positive correlation between time mastication time and the number of performed mastication cycles, regardless of oral and prosthetic status. There have been observed somewhat higher values for all types of food for chewing time and the number of masticatory cycles for patients with complete dentures or overdentures. These are probably due to the biomechanics of dentures and the morphological and functional changes of the oral structures involved, but also in relation to the age of the patients, the evolution of their oral and general status. In fact, in the case of carrot mastication, there was a tendency for recording higher time values and a more significant number of mastication cycles in those with generally altered health status and those with advanced age.

The aspects highlighted in this research can also be correlated with the masticatory force developed by the muscles during mastication, which changes with respect to age, general health, individual characteristics, and oral status. Thus, the masticatory force of the complete prosthesis bearer is considerably lower than that of the patient with his teeth, at an approximate level of only 20–40% of the former [4].

Even if a completely edentulous patient is orally rehabilitated with the optimal prosthesis, the efficiency and masticatory force will be well below the values of the dentate or fixed prosthetic patient. Helkimo et al. [5] and Laurence Mioche et al. [6] reported the decrease of the masticatory force with age, which manifests itself more in women than in men. Michael et al. [7] concluded in their study that the closing force in occlusion and mastication is different between dentate and edentate, and complete denture wearers have a disability when it comes to masticatory force. These results are consistent with those of our research, which suggests that, in the case of the dentate, the parameters of mastication show superior characteristics to those encountered at the edentate. Zamacona et al. [8] concluded that the occlusion and conception of the occlusal relief of the lateral teeth of the total prostheses are decisively important for their masticatory stability and efficacy. However, the masticatory function and patient satisfaction, assessed subjectively, do not always correspond to the real, objectively assessed situation.

It is advisable under these conditions to alert the patient wearing complete denture to the changes that occur in mastication, requiring patience and effort to adapt to the new conditions. In adapting mastication to oral changes, produced by tooth loss and prosthesis, the patient goes through a learning process of mastication under the new conditions, during which already installed reflex circuits change according to the new neural conditions resulting in a motor effect adapted to new prosthetic conditions. For rapid adaptation with oral changes, it is necessary to create – at an oral level – normal conditions of the already formed reflexes. The volume, the shape of the prosthetic parts, must fit in the functional space resulted from the loss of the teeth (the neutral area), and the morphology and occlusal teeth encounter, together with the established inter-maxillary relations (as vertical dimension of occlusion) should allow the correct closure of the neural reflexes [9–11]. Discomfort and oral pain can cause new, incorrect reflexes that can become permanent and harmful [12]. Practical experience is indicating that the instability of complete dentures, a relatively common aspect contributes significantly to masticatory dysfunction; therefore, it is recommended to review alternative treatments, for example with implant placement, including mini dental implants with a positive role on patient satisfaction and quality of life [13–15].

Changes in the general state of health may have various local and systemic effects, possibly about associated medication as well [16,17]. Among these undesirable consequences is the impairment of the masticatory function, which is supported by the results of this research.

Food consistency, their degree of adherence or saliva absorption capacity can influence the process of chewing and, implicitly, swallowing within digestion. Feldman et al. [18] and Peyron et al. [19] argue that the number of mastication cycles to achieve mastication increases progressively with age, which is supported by this study, which found a direct proportional relationship between increased age and number of mastication cycles, but only in the case of carrot mastication. Regarding the chewing frequency, there were differences concerning the type of food, differences between dentate and edentate in the case of mastication of the apple, and among those with a clinically healthy overall status and those with general alterations in the case of apple. These results are consistent with other research, such as the one attained by Bessadet et al. [3], which consisted of differences in chewing frequency between various types of foods, and brings arguments for the difference observed in patients with various types of edentulism, this parameter indicating a disruption of the masticatory function in a strong relationship with tooth loss.

## Conclusions

It takes different time and number of mastication cycles to complete chewing, in relation to individual and food characteristics, to the systemic, oral and prosthetic status. The number of remaining teeth and the type of prosthetic rehabilitation favor the adaptation and improvement of masticatory parameters and can have marker value for masticatory efficiency.

The time and number of chewing cycles until swallowing of the food bowl are indicators of the functional state of the masticatory system and the level of maximum bite force resulting from the combined action of the jaw lift muscles modified by jaw biomechanics and reflex mechanisms.

The duration of mastication, the number, and duration of mastication cycles, the frequency of mastication as dynamic objective parameters are important indicators of the masticatory efficiency and the level of functional adaptation on prosthetic rehabilitation, which can be correlated with the patients’ perception and satisfaction.

## Conflict of Interest

The authors confirm that there are no conflicts of interest.
